# Address

**Published:** 1877-04

**Authors:** E. J. Waye


### DENTAL REGISTER.

VOL. XXXI.] APRIL, 1877. [NO. 4.

### ADDRESS.

BY E. J. WAYE, D. D. S.

*To the Graduating Class, Ohio Dental College, March 7, 1877.*

GENTLEMEN OF THE GRADUATING CLASS:—The present occasion is one to which you have all doubtless looked forward with eager anticipation, not unmixed with a degree of anxiety. It has been in your thoughts during the hours of preparation and study, through which you have passed, stimulating you to earnest and persevering effort, and beckoning you through its bright portals into the noble profession of your choice. The first great object for which you have struggled is to-night accomplished, and as with retrospective glance you scan the toilsome way through which it led. As your hearts are stirred with the cordial greeting of men long eminent in the profession. As they welcome you into their companionship and while the sweet intoxication of success woos you to cheerful views of the future, presenting to your dazzled gaze a vision of long life, success, distinction and eminence in your profession; it will not be strange, I think, should you come to regard the toil and study through which you arrived to the present honor as things of the past, no longer to be dreaded or endured, and only remembered as the tiresome, though necessary preparation for the new life opening before you, and to regard the future as a broad sunny path ever widening in beauty and loveliness as it leads onward to honor and success.

It is no part of my mission to-night to darken this bright picture of the future, but rather, so far as in me lies, to offer such advice and suggestions (the fruit of a life-long experience) as, if followed, even though they fail to bring the full realization of your glowing fancies, yet may at the least tend to make the path you have chosen less rugged, and the obstacles which you will inevitably encounter less insurmountable. In all that pertains to preparation for the life of a dentist, it is assumed, and believed you are in no degree deficient.

The diploma of the Ohio College of Dental Surgery, an institution, which in the extent of its curriculum, and the thoroughness of the methods of teaching, is believed to be second to none in this country, is to-night conferred upon you; a fact, which in itself, I consider the best proof that in all that knowledge which constitutes the groundwork of the profession, you are so far as correct theories and thorough teaching can make you, fully competent to practice intelligently and successfully the duties of our noble profession.

As students under the supervision and instruction of professors and teachers, your career is to-night ended. You are now to enter upon a new domain, the domain of actual practice. In the new role to which you are promoted, the knowledge so obtained is to be put into practical service.

In the past, when perplexed by difficulties seemingly unsurmountable, the teacher has been at hand to lend his ready aid in their solution. Henceforth you are to practice what you have acquired; alone and unaided you are to minister to the demands of suffering humanity. In your skill and knowledge it confides, and wo to you should you be found wanting. You will be expected to form a correct diagnosis of every varying case presented; to adopt the proper treatment to restore to health and usefulness the diseased and painful organs. The nervous, the timid, the fidgety and the unreasonable, each and all will challenge your skill, patience and forbearance, and while demanding your highest exercise of each, will at the same time, perhaps, question your ability or understanding of the case. The well being of your patients—nay, your own reputation and usefulness requires that these various demands upon you shall be intelligently, promptly and successfully met, and all this at the threshold of practice.

It is, as I have said, no part of my mission to discourage. It should be no discouragement to a true man to be fully informed beforehand of the difficulties and trials he is to encounter. It is this very knowledge which by affording the opportunity for preparation should tend to make success the more certain. "Forewarned is forearmed," is an old saying full of sense and wisdom, and he who being forewarned is not found prepared, is not a man from whom we should expect great things; but the trouble in this particular case, lies in the fact that though you may understand beforehand what is needed, you can not at once obtain it.

You enter professional life, as indeed every man has and does, lacking a certain most essential element of successful practice, viz: experience, and what is a peculiarly embarrassing circumstance connected with it is the fact that this same experience can only be acquired in that school of daily practice, in which it is so important an adjunct. At the outset when most needed it is entirely withheld, and only when long practice would seem to have rendered its aid unnecessary, has its treasures of wisdom become your own. But fortunately for you and every other man who enters upon life, while each has his own personal experience to pass through, by which he will profit according as circumstances or his own tact in moulding them to his own advantage shall determine; the knowledge thus acquired is not necessarily confined to himself; it may be imparted to a friend, or published to the world, and thus mankind are enabled in a measure to substitute it for their own, when as tyros they enter upon whatsoever business or profession. "Experience," says Franklin, "is a dear school, but fools will learn in no other." That is to say the experience which is founded upon ignorance, though it may be both sound and practical, must of necessity be also limited in scope and tedious in acquirement. It is that experience which waits upon knowledge that is truly the most valuable and instructive; not as a means for the acquisition of knowledge but rather as a wise judge pronouncing upon it, and with unerring wisdom verifying and approving the true and detecting and condemning the false is its teachings of the greatest possible value to him who will profit by them. But the maxim contains another clause and another truth, stated with the philosopher's usual terseness and perspicacity: "We may give advice but we can not give conduct." What signifies it though the advice be most valuable and instructive, the result even of a life spent in its acquisition, if no man should harken or profit by its wise counsels? My friends, experience is like the chart which the mariner upon the broad ocean, in search of unknown shores with naught beside to direct him, studies with anxious care and carefully shapes his course in accordance with its lines, the records of another's voyage. And though his vessel be staunch and seaworthy, his crew trusty and tried, his outfit in all respects complete, and his knowledge of every detail perfect, without that guiding chart his course at best must be but devious and uncertain, and if after weary days and sleepless nights he at length reaches the port of his destination, it is more the result of blind chance than of wise forethought or prudent calculation. In the untried realm of practice no such chart is available, the most that is allowed is a staunch vessel and a sturdy and disciplined crew; with these, your compass, books and general outfit, you are to lift anchor and strike your course. The channel may be tortuous, the tide unfavorable, sunken rocks and treacherous quicksands may threaten and obstruct your passage, but while you pause in doubt and uncertainty, a small boat glides alongside and the pilot's cherry hail falls upon your ear—gladly you place the helm in his experienced keeping and your voyage is auspicously begun. The experience of another is to guide you through the unknown and hidden dangers of the start, and when at length an open sea, unclouded skies and a favoring breeze shall again inspire you with hope and courage, he yields the gallant vessel again to your control, and bidding you God speed, leaves you in safety and security upon your onward pathway.

It might be inferred from my having dwelt with such a wealth of proverb and metaphor upon the advantages to be derived from experience that there may be much in my own, somewhat, which I deem of sufficient importance to warrant enlarging upon it, and while my vanity might prompt an affirmative reply, I am still forced to check it, while I declare that the experience of no single individual, while there may be much in it of value, is to be compared for one moment with the great aggregate which is in these days at the man's service, who will lend a tolerably attentive ear, and in this respect there is a most marked contrast to the long ago when I first essayed the dentist's art. In those days, advice and experience were valuables not quite so freely bestowed. There were at that time "secrets which no fellow could find out." He was indeed but a very common place dentist whose stock of knowledge did not include several, each and all, of course, uncommunicables. Therefore was it that men trusted each to his own experience, and evolved from his own inner consciousness, such wisdom and knowledge as could be gleaned from that overworked and not usually fruitful field. Of books there were few. Harris was not, or might as well have been for any knowledge I posessed, and the Dental News Letter was as yet a dead letter. The dental library of my preceptor was at once brief and comprehensive. It consisted of a single volume of three hundred pages, or thereabouts. Title, Fox's Mawry. It contained a little anatomy, do physiology, do pathology; some account of the materials and instruments used in filling teeth. Any time within a hundred years previous to Harris, fully one-half the volume was devoted to mechanical dentistry, and for the time was fairly full and comprehensive; with a few illustrations. I may without vanity say that in the two years I remained, I had nearly mastered this epitome of dentistry, and with the added experience of the laboratory, (so termed per courtesy), and much clinical instruction in the extraction of teeth, chiefly by the aid of the turnkey, I was at the end of this time bidden God speed, with the assurance, with impressment, that save in the single item of experience, I knew as much as my preceptor. This I considered at the time as the highest commendation; but I have since had occasion to modify my views somewhat, indeed, within a comparatively short period I arrived at the conclusion, either that my preceptor himself had some things yet to learn, or that my own experience was sadly deficient; if the latter, it has (I may say without boasting), since, been in a measure remedied. From this brief retrospect it will appear that at that time the study of the dental art was the "pursuit of knowledge under difficulties," and although its requirements were neither varied or extensive, the acquisition was attended with much that was unfavorable to the development of a noble and generous professional sentiment. It was the instruction of the laboratory and extracting chair. The isolation and jealousy which characterised this era of the "secret," the lack of all opportunity for comparing his own operations with those of others, tended to foster a spirit of conceit and egotism, which being reflected in turn upon the student, engendered in him also the same intolerant notion of his own superiority, which only association with other men, and a comparison of attainments could effectually dissipate. It is this comparison of ourselves with others, by which only we may reach just conclusions; and though in arriving at them we may sometimes find our self-esteem abated very considerably and a degree of mortification may ensue, yet it will usually be found that when this occurs, that our conceit will very well bear pruning, and that the correcting process while bringing us down from our too elevated position to the true level, has at the same time accomplished another result almost as important, viz: that of exalting a professional brother from our too humble estimate to his actual standing. This done we are prepared for that generous emulation, out of which grows every good resulting from professional intercourse. And in this connection I would remark that the wonderful advancement in the profession which followed the movement of association, and has resulted in making of dentists throughout the world, the great brotherhood which they now are, is believed to be attributed to the friendly interchange of views, thoughts and ideas from that true standard of estimating them, viz: of comparing ourselves with others. It would be interesting, did time permit, to trace briefly the leading influences which brought us up from the doubtful professional status of that period, to our present recognized standing as a leading and independent medical specialty. Among the many potent influences which contribute thereto, earliest and most effective, I think we may place the dental college whose graduates understanding the intimate relation existing between the masticatory and the digestive apparatus and appreciating the importance of those "pearls without price," which were being ruthlessly sacrificed to give place to artificial dentures, in no respect to be compared with them, either as to beauty or effectiveness, were stimulated to more strenuous efforts for their preservation.

The discovery of the cohesive property of gold, and with it the possibility of restoring lost portions of teeth, not accomplished previously, added impetus to their zeal. New instruments and appliances assisted, and when to this was added associated effort in the way of societies, local, state and national, which became in time, clinics, in which the finest operations could be witnessed by every member of the profession, whose curiosity, if no deeper motive, prompted him to be a spectator. Nearly every man felt an ambition to produce equally beautiful fillings, and ere long, elegantly finished operations in contour fillings became the popular idea; and those who performed them were rated in the profession *above* the more studious and cautious investigators whose carefully conducted experiments in the study of nerve, tissue or cell, had well prepared them to test understandingly the organs entrusted to their care. These men may have failed to make fillings with so perfect a finish, but as you are aware my friends, merely elegant fillings, without the knowledge when, and under what circumstances to insert them, will hardly succeed in the long run; and now it is beginning to be very generally understood that it is the two things—knowledge combined with manipulative ability which make the dentist, and neither alone will succeed.

You are entering the profession at a time when to be successful will demand your highest efforts; for your encouragement it may be added, that such efforts will be generally appreciated, and the respect and patronage you merit will be bestowed.

As the result of an experience which in many essentials is common to all who enter the profession, and to the end that you may be spared some of the unpleasant lessons of that severe school, I offer, by way of closing, a few brief suggestions, which if adopted at the outset may possibly serve as land marks to aid in directing your course.

The dentist should be eminently a conscientious man.

Of no one thing, I am confident, so common and important, does there exist so dense an ignorance as of the teeth, in a general sense. Most, or at least a great many people, only arrive at a realizing sense of their posession, when by neglect and subsequent decay they cause pain. Such persons come to you in distress, and entire ignorance of what is the proper thing to be done; in such a case, if your manner be such as to inspire confidence, your advice will be sought and probably accepted. Put yourself in the place of the patient; carefully consider all the circumstances, and asking, what with your knowledge of the case, you, in his place, would desire to have done; advise and treat accordingly,

Be equally conscientious in performing all operations in the best possible manner, even to the minutest detail.

Cleanliness. See to it that all your instruments and ap pliances of every kind are scrupulously clean. Many of your patients will be ladies, whose sense of refinement and delicacy is the most exquisite. Remember that you operate within the mouth; that many of your instrument come in contact with the lips, and that the hands are continually subject to the closest scrutiny,

The most scrupulous attention to cleanliness will therefore not be unworthy your attention.

Patience. Never, if possible, lose your temper, however lacking in decision and fortitude your patient may appear; do not forget that many will have suffered excruciatingly long before seeking your advice and assistance; and also that their nervous organization may be more sensitive than you imagine.

Do not by your apparent insensibility and lack of sympathy, wound or drive away patients whose gratitude and regard it is worth your while to cultivate, and whose influence may bring you appreciative patrons.

Dentists have been heard to say that it was no part of their concern how much pain they inflicted. To those who indulge in such expressions, in the hearing of patients, I would suggest that they will need to be very superior operators to induce persons of sensitive nerves to place themselves under their care.

Promptitude. Be prompt to fulfill your own promises and engagements, and there will be the more reason for exacting the same from others. Intelligence and refinement should characterize every dentist ambitious of success, and the more cultivation the better.

Be manly and courteous in your relations with others, refined and dignified in demeanor, honest and honorable in all things, studious and observing, with a habit of thinking and recording.

And now in conclusion I will only say that your future is most inspiring, entering the profession at this most auspicious era of prosperity through the portals of its highest institution, the dental college, surrounded by the ripe fruits of other's toil and experience, which you have but to gather and add to the hard earned knowledge already garnered.

What expectations are too sanguine of the incoming generation of dentists? Great as has been the advancement in the past, the future must far exceed it.

The past has indeed had its giants in the profession, but you—you young men are above them. You stand upon their shoulders, the wisdom and experience of the century past is yours, not as with us, the older members, as we are quitting the stage, but even at the entrance, we can but cheer you on the upward path, while confiding to your warm hearts and honest and earnest efforts, the future of our loved profession.

